# Solid screw insertion for tension band plates: a surgical technique tip

**DOI:** 10.1007/s11832-016-0748-2

**Published:** 2016-06-16

**Authors:** Muayad Kadhim, Ahmed I. Hammouda, John E. Herzenberg

**Affiliations:** Rubin Institute for Advanced Orthopedics, Sinai Hospital of Baltimore, 2401 West Belvedere Avenue, Baltimore, MD 21215 USA; Department of Orthopedic Surgery, Al-Azhar University Hospitals, Cairo, Egypt

**Keywords:** Tension band plate, Growth modulation, Bone screws

## Abstract

**Purpose:**

Growth modulation with tension band plates (TBPs) and cannulated screws is the current mainstay of treatment for pediatric lower extremity angular deformities. Solid screws have been used as an alternative to cannulated screws to decrease the risk of screw failure, particularly in obese children. The downside of solid screws is the decrease in precision of placement. This study describes a surgical technique to insert solid TBP screws accurately.

**Methods:**

TBP insertion starts with the same conventional steps by inserting the guidewires into the epiphysis and metaphysis, straddling the physis. After fluoroscopic confirmation of the position of the guidewires, the cannulated drill bit is used to broach the cortex to a depth of 5 mm in the bone. A standard 4.5-mm cannulated screw from the TBP set is used to tap the screw tract over the guidewires for approximately three-quarters of the planned screw length. After removing the guidewires, the solid screws are then inserted in each hole to follow the tapped tracts.

**Results:**

This technique was used in five patients including four with Blount disease and one with bilateral genu varum.

**Conclusion:**

It is recommended to use solid screws with TBPs in patients with a high body mass index to avoid screw fracture. Our technique describes using a cannulated screw as a tap to create a tract to ease accurate insertion of the solid screws, and prevent the solid screw from deviating outside the desired path.

## Introduction

Lower extremity angular deformities such as genu varum or valgum are common in children. Treatment options include osteotomy [1] or growth modulation [2–9]. Growth modulation is less invasive than osteotomy and has increased in popularity, particularly since the development of tension band plates (TBPs ) by Stevens [8]. TBPs function as a hinge on the convex side of the deformity, and gradually correct limb alignment while preserving natural growth, with no need for invasive osteotomy [4, 8].

TBPs use cannulated screws which provide ease and accuracy of screw insertion over a guidewire [3, 5, 8]. After first passing a 1.5-mm guidewire, a 3.2-mm cannulated drill is used to broach the cortex to a depth of 5 mm followed by insertion of cannulated 4.5-mm screws. Such accuracy is very important especially in young children to avoid injuring the physis and to avoid penetrating into the joint. Several reports have described broken screws in TBP constructs, particularly in obese patients [5, 10, 11]. In response to this problem, the manufacturers are now offering solid, non-cannulated screws for added strength and resistance to fracture.

We consider using solid screws for obese patients, as is commonly seen in adolescent Blount disease. The downside of solid crews is that they cannot be placed with the same high degree of precision as cannulated screws. The senior author developed a technique to insert solid screws into TBPs, while still maintaining the accuracy of the cannulated system.

### Surgical technique

The surgical technique for inserting a TBP is similar to the standard technique described by Stevens [8]. The image intensifier is placed at the opposite side of the surgical field. A 2-cm longitudinal skin incision is made at the physis center to expose the periosteal surface. The central hole is used to center the TBP over a guide pin or a 20-gauge spinal needle that is inserted into the physis.

The epiphyseal and metaphyseal guidewires are then inserted through the eccentric holes by using the supplied drill guide, under fluoroscopic control for accurate placement straddling the growth plate. Two pilot holes are then drilled using a 3.2-mm cannulated step drill bit to a depth of 5 mm. The drill bit should not be drilled deeper than 5 mm, as this removes bone, weakens screw purchase and deceases pull-out strength.

**Fig. 1:**
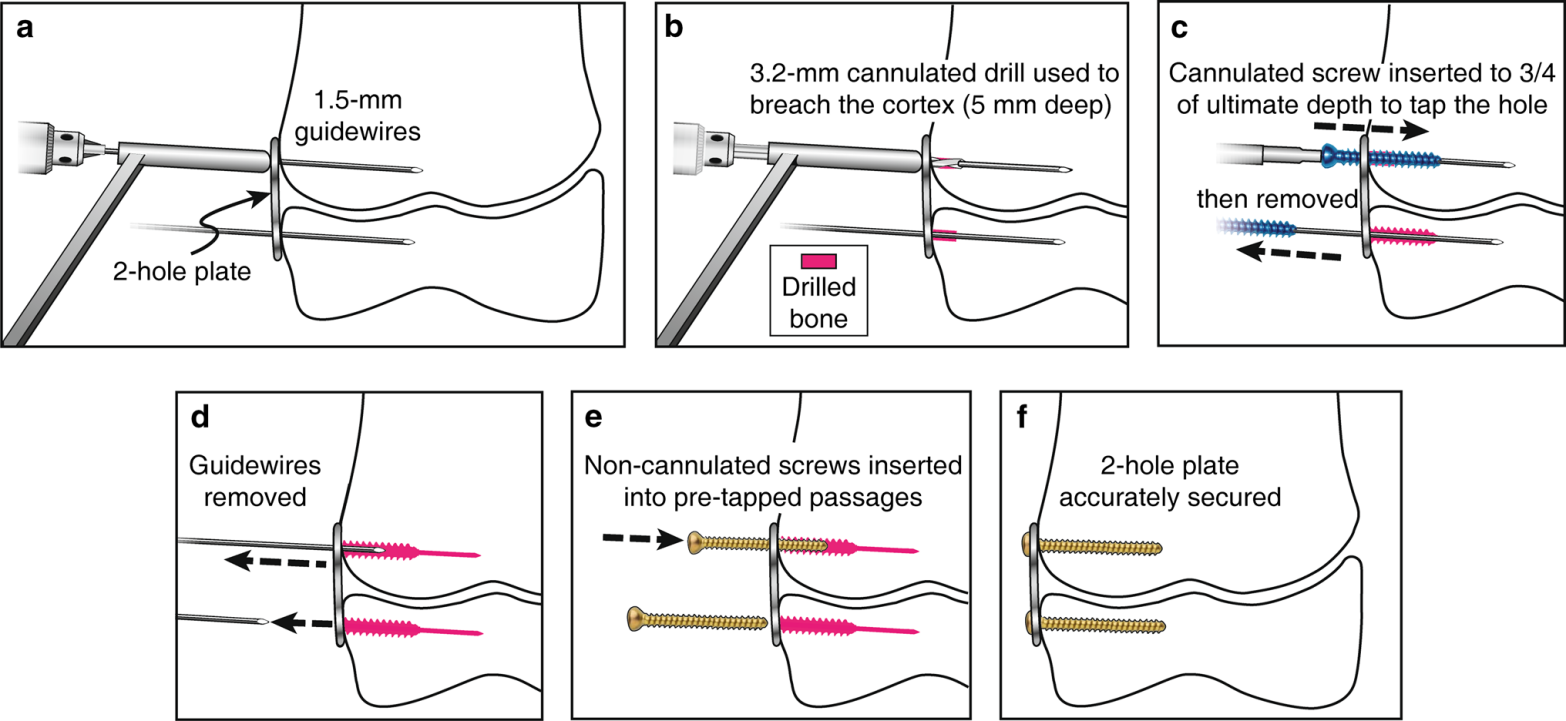
The surgical technique for TBPs with solid screws. **a** A K-wire is inserted in each of the plate holes, **b** a 3.2-mm cannulated drill bit is used to broach the cortex no more than 5 mm depth, **c** a cannulated screw is inserted to tap three-quarters of ultimate depth, **d** the guidewires are removed, **e** the solid screws are inserted into the pre-tapped passages, **f** the plate is secured with two solid screws. Copyright 2016, Rubin Institute for Advanced Orthopedics, Sinai Hospital of Baltimore

**Fig. 2:**
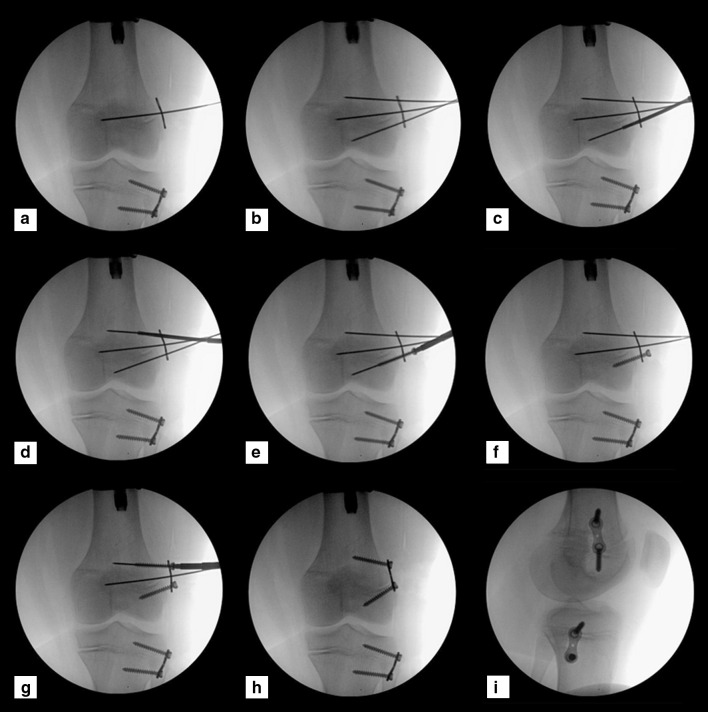
Intraoperative images demonstrating the surgical technique. **a** A central K-wire is inserted at the physis level, **b** a K-wire is inserted in each eccentric screw hole of the plate, **c, d** a 3.2-cannulated drill bit is used to broach the cortex at each screw hole, **e** a cannulated screw is used as a tap, **f** a solid screw is inserted into the distal hole, **g** a cannulated screw is used to tap the proximal hole, **h, i** follow-up films during correction. Copyright 2016, Rubin Institute for Advanced Orthopedics, Sinai Hospital of Baltimore

A 4.5-mm cannulated screw is then used as a tapping tool over the guidewire to tap at least three-quarters of the desired screw length. The guidewire is then removed to insert a 4.5-solid screw that follows the path created by the tapping (Figs. 1, 2). Controversy exists regarding parallel or divergent position of the two screws.

## Materials and methods

**Fig. 3:**
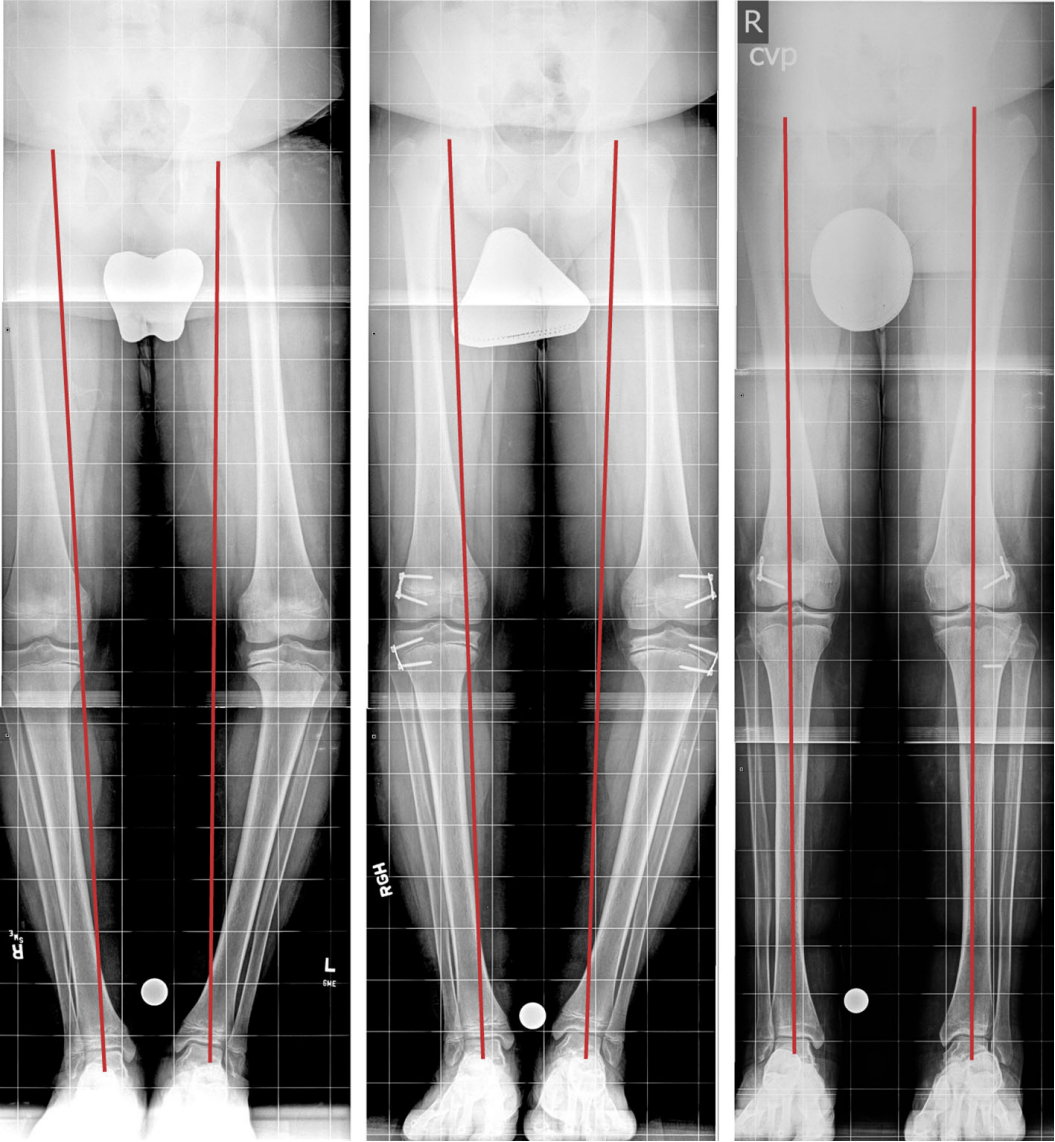
Twelve-year-old male with bilateral Blount disease, underwent bilateral distal femoral and proximal tibial lateral hemiepiphysiodsis with a TBP. **a** Long erect X-ray shows preoperative bilateral varus deformity with medial mechanical axis deviation, **b** 3 months after insertion of bilateral distal femur and proximal tibia lateral physeal TBP, **c** last follow-up with corrected deformity and central mechanical axis. The broken left tibial metaphyseal screw occurred 2 years earlier, when the first TBP was inserted for gradual varus correction. Copyright 2016, Rubin Institute for Advanced Orthopedics, Sinai Hospital of Baltimore

Solid screws have been used with TBPs since 2008 in five patients, including four with Blount disease and one with bilateral genu varum. The five patients (two male, three female) had a mean age at surgery of 12.1 years (range 7.2−14.6 years). Figure 3 demonstrates a 12-year-old male with bilateral Blount disease who had bilateral distal femoral and proximal tibial lateral hemiepiphysiodsis. A TBP was inserted using two solid screws using the same insertion technique.

## Discussion

The rationale behind temporary growth modulation for lower limb angular deformity is to allow for gradual correction while the physis is still growing and avoid the need for invasive osteotomy. Excellent results have been reported with the use of TBPs; however, fractures of the cannulated screws have been reported, mainly in obese patients with Blount disease [5, 9–11].

Burghardt et al. proposed two modifications in order to avoid hardware failure. The first is to apply two TBPs side by side and the second is to use solid screws rather than cannulated screws [10]. Solid screws are stronger and bear the mechanical stresses more than cannulated screws [12]. The mechanical strength of a screw depends on the core diameter. Therefore, cannulated screws are weaker to torsion and bending forces compared to solid screws [13]. Stitgen et al. studied two different TBP constructs and concluded that solid screws are stronger than cannulated screws [14].

The disadvantage of solid screws is that they are harder to accurately insert than cannulated screws. Here, we describe our technique to insert a solid screw into a TBP with ease and accuracy similar to cannulated screws. The surgical technique is consistent with that described by the manufacturer who recommends pre-drilling only 5 mm into the cortex, i.e., not all the way through the bone. The rationale for this is to increase the screw purchase within the bone, especially the metaphyseal cancellous bone. For this reason we do not drill the entire screw length when a solid screw is used. Instead, we use a cannulated screw to tap the screw trajectory over the guidewire. The ‘screw’ tap pushes the bone aside and impacts it, making it more dense. In comparison, drilling with a cannulated 3.2-mm drill bit over the entire length would remove bone and result in decreased screw purchase. The initial pass with a cannulated screw creates a clean channel for the solid screw to follow and helps avoid deviation of the solid screw. The solid screw must be inserted carefully without applying extra force or pushing on the screwdriver to avoid deviating from the pre-tapped tract close to the physis. A tied suture around the screw head can be used to prevent screw displacement in the soft tissue especially in obese patients as previously described in submuscular plating techniques [15].

The fate of the extra cannulated screw which is used as a tap may depend on where you practice. In the USA, it is common hospital practice to discard any screw that is inserted into the body, even if it is immediately removed. This is due to concern that the screw may be weakened by insertion, e.g., if it is rubbing against the side of a plate during insertion. This practice is common in the USA during open reduction internal fixation of long bone fractures; a screw that is inserted and found to be too long or too short, is commonly discarded and replaced. However, in other parts of the world, this practice is less common, and a screw that is inserted briefly and removed can be recirculated into the tray to be used for another case. A third option is to select one long cannulated screw to be used as the ‘tap screw’ and keep it separate from the implant screws along with the instrument part of the tray, to be used over and over again, in a similar way as a tap is repeatedly used. Finally creating a special instrument (tap) supplied by the company is a good concept that could be considered by the instrument manufacturers.

There is some controversy about the optimum position for screws in guided growth. At least one study argues for initial parallel placement [16]. However, on a practical note, it is not always possible to anatomically get the screws parallel as the growth plate is undulating, and the surface of the bone is sloped. Therefore, for practical issues, it is often necessary to insert the screws divergently. Regardless of where you stand on this issue, the purpose of this study is not to advocate for parallel versus non-parallel screw insertion, but rather to describe a technique that allows accurate placement of non-cannulated screws according to the wishes of the operating surgeon and the specific anatomic considerations of the individual case.

In this study we describe a surgical technique to use a cannulated screw as a tap to clarify the desired screw trajectory prior to solid screw insertion. We believe the use of a tapping screw is better than drilling the full trajectory with a 3.2-mm drill bit, to increase the screw purchase with the cancellous bone.
